# Vitamin C Is Essential for the Maintenance of Skeletal Muscle Functions

**DOI:** 10.3390/biology11070955

**Published:** 2022-06-23

**Authors:** Shoko Takisawa, Yuka Takino, Jaewon Lee, Shuichi Machida, Akihito Ishigami

**Affiliations:** 1 Molecular Regulation of Aging, Tokyo Metropolitan Institute of Gerontology, Tokyo 173-0015, Japan; aime.mu2ic@gmail.com (S.T.); yuka.t915@gmail.com (Y.T.); 2 Department of Pharmacy, College of Pharmacy, Pusan National University, Busan 46241, Korea; neuron@pusan.ac.kr; 3 Graduate School of Health and Sports Science, Juntendo University, Chiba 270-1695, Japan; machidas@juntendo.ac.jp

**Keywords:** ascorbic acid, muscle atrophy, physical ability, SMP30, vitamin C

## Abstract

**Simple Summary:**

Previously, we reported that long-term vitamin C (L-ascorbic acid, VC) deficiency causes muscle atrophy and deterioration in physical ability using female senescence marker protein-30 (SMP30)-deficient mice with a lack of VC synthesis, which is similar to that observed in humans. In this study, we investigated whether these findings also hold true in male SMP30-deficient mice and found that long-term VC deficiency causes muscle atrophy and deterioration in physical ability in male SMP30-deficient mice as well. Interestingly, muscle atrophy and declined physical ability were completely restored with VC supplementation. Thus, VC is essential for maintaining skeletal muscle function in both male and female mice.

**Abstract:**

Vitamin C (L-ascorbic acid, VC) is a water-soluble antioxidant essential for collagen polymerization. Previously, we reported that long-term VC deficiency causes muscle atrophy and deterioration in physical ability using female senescence marker protein-30 (SMP30)-deficient mice with a lack of VC synthesis, which is similar to that observed in humans. To determine whether these findings also hold true for male SMP30-deficient mice, two-month-old male SMP30-deficient mice were divided into two groups: the VC-treated group (VC(+)) was administered 1.5 g/L VC, and the VC-untreated group (VC(−)) was supplied water without VC. The VC level at four weeks in the gastrocnemius muscles from the VC(+) and VC(−) groups was 205.7 ± 8.5 nmol/g tissue and 13.1 ± 0.6 nmol/g tissue, respectively. Thus, four weeks was enough to reduce the VC level in the skeletal muscle in the VC-untreated group. On the other hand, muscle weights of the gastrocnemius, soleus, plantaris, tibialis anterior, and extensor digitorum longus in the VC(−) group were significantly reduced by VC deficiency after twelve weeks. The physical endurance of the VC(−) group at eight weeks was markedly lower than that of the VC(+) group. The grasping strength and activity in the cage in the nocturnal phases of the VC(−) group were markedly lower at twelve and sixteen weeks than those of the VC(+) group. Interestingly, muscle atrophy and declined physical ability were completely restored with VC supplementation for twelve weeks after VC deficiency. Thus, VC is essential for maintaining skeletal muscle function in both male and female SMP30-deficient mice with a lack of VC synthesis.

## 1. Introduction

Vitamin C (L-ascorbic acid, VC) is a water-soluble antioxidant that scavenges reactive oxygen species such as hydroxyl radicals, singlet oxygen, and superoxide radicals [1,2,3,4]. Under physiological pH conditions, VC most commonly exists in its monoanion form, ascorbate [5]. VC also acts as a cofactor for enzymes that are important in the construction of collagen fibers, which are abundant in the skin and cartilages [6,7], the metabolism of lipids such as cholesterol [8], and the synthesis of catecholamine such as adrenaline [9]. Many vertebrates have the ability to synthesize VC [10,11]. However, humans, nonhuman primates, and guinea pigs cannot synthesize VC because of multiple mutations in the *Gulo*-encoding gene L-gulono-γ-lactone oxidase, the last enzyme in the VC synthesis pathway [12,13].

Senescence marker protein-30 (SMP30) has been shown to decrease in the liver, kidneys, and lungs of aged mice [14]. In addition, SMP30 is a gluconolactonase, catalyzing the reaction of L-gulonic acid to L-gulono-γ-lactone in the VC synthesis pathway [15]. Therefore, SMP30-deficient mice lack VC synthesis, which is similar to what has been observed in humans.

Human skeletal muscle comprises approximately 40 % of total body weight and contains 3–4 mg of ascorbate per 100 g in skeletal muscle [16,17,18]. Previously, we conducted a cross-sectional analysis, in which 957 elderly women (70–84 years old) living in Japan participated in to investigate the relationship between plasma VC levels and exercise function [19]. The results of this study showed that the plasma VC level was significantly correlated with the subject’s muscle strength (handgrip strength), balance capability (ability to stand on one leg with eyes open), and normal walking speed. In other words, elderly women with higher plasma VC levels tended to have stronger muscle strength and physical ability. Additionally, Martin et al. [20] reported in a Hertfordshire cohort analysis of community-dwelling elderly people (348 men and 280 women) aged 63–73 years in the United Kingdom that elderly women with a high VC intake had higher physical function such as a significantly shorter time to stand up from a chair. However, elderly men with a high VC intake did not have higher physical function. Thus, older women with higher plasma VC levels and a high VC intake are believed to have higher muscle strength.

A sex-based difference in VC requirement has been suggested by several studies. Levine et al. [21] examined the association between the dose and steady-state plasma VC level in young women and proposed that the recommended dietary allowance of VC for young women should be increased to 90 mg daily, while the recommended dietary allowance of VC for men is 75 mg daily. Moreover, Sasaki et al. [22] reported that the plasma VC level was higher in females than in males throughout all ages, estimated on 217 healthy controls, whose ages ranged from 12 to 96 years. Thus, VC requirements and maintenance in vivo vary with gender and age.

VC is abundant in skeletal muscles and plays an important role in the construction of collagen fibers, which are major components of tendons that connect muscles to bones [6,23,24,25]. To elucidate the function of VC in skeletal muscles as well as the effects of VC deficiency, we previously examined the effects of VC deficiency in female SMP30-deficient mice [26]. The results showed that long-term VC deficiency was associated with muscle wasting and declined bodily functions estimated using physical endurance, grasping strength, and activity in the cage, which could be recovered by restoring VC levels in skeletal muscles. However, it remains unclear whether these findings have been observed in male SMP30-deficient mice. Therefore, in the present study, we investigated whether these findings also hold true in male SMP30-deficient mice, in which VC deficiency leads to skeletal muscle atrophy and declined physical ability.

## 2. Materials and Methods

### 2.1. Animals

**Figure 1:**
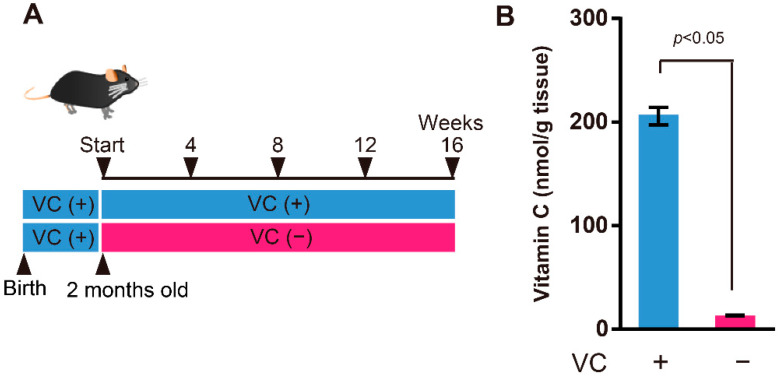
VC levels in gastrocnemius muscles. (**A**) Experimental procedure. Mice drank 1.5 g/L VC in water until two months old. Then, mice were divided into two groups: VC-treated (VC(+)) group and VC-untreated (VC(−)) group. The VC(+) group drank 1.5 g/L VC in water, whereas the VC(−) group drank water without VC for a sixteen-week period. (**B**) Total VC levels in the gastrocnemius muscles at four weeks. Values are given as the mean ± SEM of five animals. Statistical differences were considered significant at *p* < 0.05 between VC(+) and VC(−) groups.

SMP30-deficient mice were established as previously described [14]. In this study, we used male SMP30-deficient mice. Mice drank 1.5 g/L VC in water until two months of age. Then, mice were divided into two groups: VC-treated (VC(+)) group and VC-untreated (VC(−)) group, as shown in Figure 1A. The VC(+) group drank 1.5 g/L VC in water, whereas the VC(−) group drank water without VC for a sixteen-week period, after which the experiment ended. During the experiment period, all mice were housed under conditions of 22 ± 1 °C and 55 ± 5% humidity under a 12 h light/dark cycle.

For the function restoration experiment, VC(−) mice drank water without VC for twelve weeks and then drank 1.5 g/L VC for twelve weeks (VC(−)→(+)). For comparison, VC(+) mice drank 1.5 g/L VC for twenty-four weeks.

All drinking water was supplemented with 10 µM ethylenediaminetetraacetic acid (EDTA) to stabilize the VC. During experiment periods, all mice were fed a CL-2 diet (CLEA Japan, Tokyo, Japan) without VC. Skeletal muscles were collected at four, eight, twelve, and sixteen weeks, and were stored at −80 °C until use.

### 2.2. Measurement of VC Level

VC level of the gastrocnemius muscle was determined by using high-performance liquid chromatography (Nihon Waters, Tokyo, Japan) and an electrochemical detector according to the methods described previously [15].

### 2.3. Behavioral Tests

Mice were handled for 5 min for three consecutive days before undergoing the series of physical ability tests described below.

#### 2.3.1. Physical Endurance

Physical endurance was evaluated using a treadmill, as described previously [26].

#### 2.3.2. Grasping Strength

Grasping strength was evaluated using a wire hanging chamber, as described previously [26].

#### 2.3.3. Activity in the Cage

Activity in the cage was evaluated using a three-point meter, as described previously [26].

### 2.4. Statistical Analysis

Data are expressed as the mean ± standard error of the mean (SEM). Significant differences between each group were analyzed with Welch’s t-test using statistical software (Prism 6, GraphPad Software Inc., San Diego, CA, USA). Statistical differences were considered significant at *p* < 0.05.

## 3. Results

### 3.1. VC Levels in the Gastrocnemius Muscles

SMP30-deficient mice were divided into two groups: the VC-treated (VC(+)) group and VC-untreated (VC(−)) group (Figure 1A). The VC level of the gastrocnemius muscle at four weeks was determined to understand the effects of VC deficiency. The VC level in the gastrocnemius muscles from the VC(+) and VC(−) groups was 205.7 ± 8.5 nmol/g tissue and 13.1 ± 0.6 nmol/g tissue, respectively, and a significant difference was observed between them (Figure 1B). Accordingly, VC deficiency for four weeks was sufficient to reduce VC in the skeletal muscles.

### 3.2. Skeletal Muscle Weight

**Figure 2:**
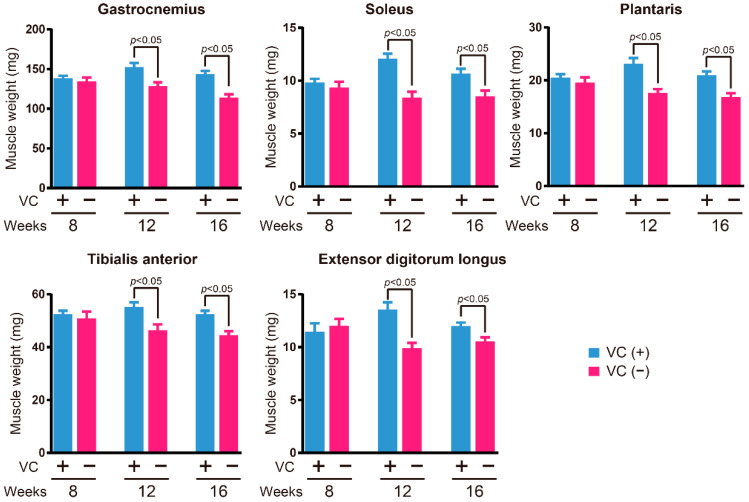
Skeletal muscle weight during VC deficiency. At eight, twelve, and sixteen weeks, *n* = 5 and 5, *n* = 10 and 10, and *n* = 9 and 15 mice each for VC(+) and VC(−) groups, respectively. Values are given as the mean ± SEM. Statistical differences were considered significant at *p* < 0.05 between VC(+) and VC(−) groups.

At eight, twelve, and sixteen weeks, major skeletal muscle weights were measured to investigate the influence of VC deficiency (Figure 2). The gastrocnemius, soleus, plantaris, tibialis anterior, and extensor digitorum longus muscle weights in the VC(−) group were significantly lower than those observed in the VC(+) group at twelve and sixteen weeks; however, no significant differences were observed at eight weeks.

### 3.3. Physical Ability

**Figure 3:**
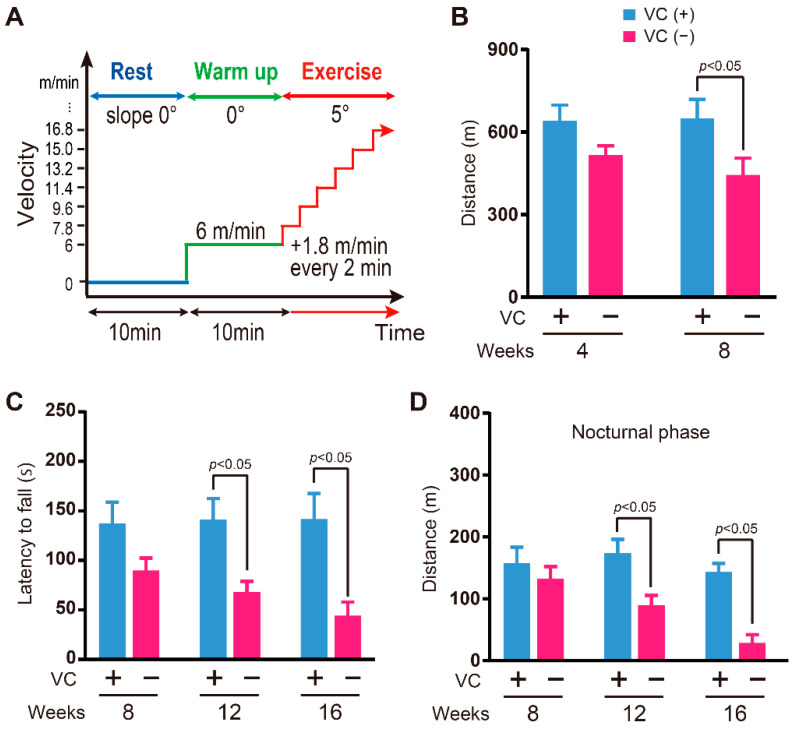
Physical ability during VC deficiency. (**A**) Running program of treadmill. (**B**) Treadmill running distance. *n* = 6 mice in the VC(+) group and *n* = 11 mice in the VC(−) group. (**C**) Wire hanging test. At eight, twelve, and sixteen weeks, *n* = 8, *n* = 20, and *n*-17 mice in the VC(+) group and *n* = 10, *n* = 25, and *n*-29 mice in the VC(−) group, respectively. (**D**) Activity in the cage. At eight, twelve, and sixteen weeks, *n* = 8, *n* = 8, and *n* = 8 mice in the VC(+) group and *n* = 8, *n* = 8, and *n* = 3 mice in the VC(−) group, respectively. Values are given as the mean ± SEM. Statistical differences were considered significant at *p* < 0.05 between VC(+) and VC(−) groups.

Since VC deficiency resulted in reduced skeletal muscle weight, we next assessed the physical ability. Physical endurance was evaluated by using a treadmill (Figure 3A). The physical endurance of the VC(−) group was significantly lower at eight weeks than that of the VC(+) group, but a significant difference was not observed at four weeks between the VC(−) and VC(+) groups (Figure 3B). The grasping strength of the VC(−) group was significantly lower than that of the VC(+) group at twelve and sixteen weeks, but a significant difference was not observed at eight weeks between the VC(−) and VC(+) groups (Figure 3C). Activity in the nocturnal phases for the VC(−) group was significantly lower than that of the VC(+) group at twelve and sixteen weeks, but a significant difference was not observed at eight weeks between the VC(−) and VC(+) groups (Figure 3D).

### 3.4. Functional Restoration

**Figure 4:**
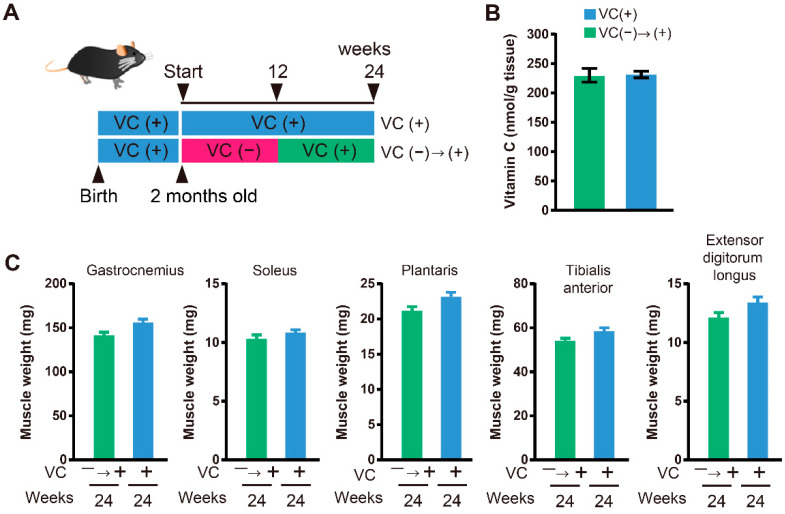
VC supplementation reversed muscle atrophy. (**A**) Experimental procedure. Mice drank 1.5 g/L VC in water until two months of age. Then, mice were divided into two groups: VC-treated (VC(+)) group and VC-untreated (VC(−)) group. VC(−) mice drank water without VC for twelve weeks and then drank 1.5 g/L VC for twelve weeks (VC(−)→(+)).VC(+) mice drank 1.5 g/L VC for twenty-four weeks. (**B**) VC levels in the gastrocnemius muscles at twenty-four weeks. *n* = 8 mice in the VC(+) group and *n* = 10 mice in the VC(−)→(+) group. (**C**) Skeletal muscle weights at twenty-four weeks. *n* = 8 mice in the VC(+) group and *n* = 10 mice in the VC(−)→(+) group. Values are given as the mean ± SEM. Statistical differences were considered significant at *p* < 0.05 between VC(+) and VC(−) groups.

**Figure 5:**
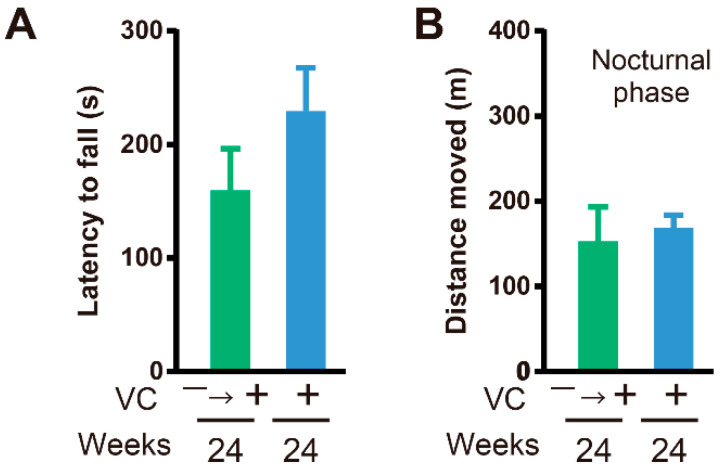
VC supplementation restored physical ability. (**A**) Grasping strength of the VC(+) and VC(−)→(+) groups at twenty-four weeks. *n* = 8 mice in the VC(+) group and *n* = 10 mice in the VC(−)→(+) group. (**B**) Activity in the cage. *n* = 6 mice in the VC(+) group and *n* = 8 mice in the VC(−)→(+) group. Values are given as the mean ± SEM. Statistical differences were considered significant at *p* < 0.05 between VC(+) and VC(−) groups.

Reduced skeletal muscle weight and declined physical function were recognized in male SMP30-deficient mice with VC deficiency. For the functional restoration study, enough VC was given to mice in the VC(−) group beginning at twelve weeks until twenty-four weeks (VC(−)→(+)), to confirm whether VC deficiency was indeed responsible for the observed effects on skeletal muscles (Figure 4A). Interestingly, the low VC level, as well as lower skeletal muscle weight caused by VC deficiency, was recovered with VC supplementation for twelve weeks (Figure 4B and Figure 4C). At twenty-four weeks, the VC level in the gastrocnemius muscle from the VC(+) and VC(−)→(+) groups was 253.8 ± 14.7 nmol/g tissue and 230.0 ± 11.6 nmol/g tissue, respectively, and a significant difference was not observed between them (Figure 4B). Moreover, physical ability, including the grasping strength and activity in the cage, in the VC(−)→(+) group was restored to almost the same levels, and a significant difference was not observed between the VC(+) and VC(−)→(+) groups (Figure 5A,B).

## 4. Discussion

This study showed that long-term VC deficiency led to muscle atrophy and declined physical ability, such as physical endurance, grasping strength, and activity in the cage in the nocturnal phases, in male SMP30-deficient mice with a lack of VC synthesis, which is similar to humans. Furthermore, VC supplementation completely restored muscle atrophy and declined physical ability.

Previously, we reported that plasma VC levels in elderly women were related to their muscle strength (handgrip strength), balance capability (ability to stand on one leg with eyes open), and normal walking speed [19]. However, it was not known whether elderly women with a deficiency in VC had lower muscle strength and physical ability. To clarify this, we previously investigated the effects of VC deficiency on skeletal muscles using female SMP30-deficient mice and found that long-term VC deficiency resulted in muscle atrophy and decreased physical ability in these mice [26]. Similarly, the present study revealed that long-term VC deficiency causes muscle atrophy and decreased physical ability in male SMP30-deficient mice as well. These results indicate that VC is essential for maintaining skeletal muscle functions. However, it remains unclear whether the presence of excessive amounts of VC in skeletal muscles results in higher muscle strength and physical ability.

In this study, four weeks of VC deficiency resulted in a reduction in the amount of VC in skeletal muscles. In contrast, skeletal muscle weight was significantly reduced only after twelve weeks of VC deficiency. Moreover, a significant reduction in physical endurance of the VC(−) group was observed at eight weeks compared to the VC(+) group, but no significant difference was observed at four weeks between them. In contrast, female SMP30-deficient mice showed a significant reduction in physical endurance after four weeks. Similar sex-based differences were also observed at eight weeks in grasping strength; that is, grasping strength showed no significant reduction in male SMP30-deficient mice, but significantly reduced in female SMP30-deficient mice. On the other hand, activity in the nocturnal phases reduced significantly after twelve weeks in both male and female SMP30-deficient mice. Thus, a reduction in physical ability due to VC deficiency occurred in both male and female SMP30-deficient mice, but the onset appeared to be earlier in female SMP30-deficient mice than in male SMP30-deficient mice.

The sex-based differences in male and female SMP30-deficient mice were presumably due to differences in VC retention in skeletal muscle, skeletal muscle weight, and/or hormonal balance and control. In fact, Tiidus et al. [27] showed that male rats had significantly higher VC levels in the plantaris muscle than female rats, although tissue VC levels were generally unaffected by acute exercise in either gender. Panda et al. [28] also showed that VC levels in skeletal muscle reduced with aging in both male and female toads and slight sex-based differences were observed during aging. Moreover, Jiao et al. [29] reported a sex difference in the expression levels of oxidative stress genes associated with VC by genome-wide gene expression profiles using L-gulonolactone oxidase (*Gulo*)-deficient mice, which were unable to synthesize VC. Thus, there have been several reports of sex differences in skeletal muscle, but no clear conclusions regarding detailed mechanisms have been reached.

The supplementation of VC from twelve to twenty-fourth weeks sufficiently restored the VC level of skeletal muscles and skeletal muscle weights of the gastrocnemius, soleus, plantaris, tibialis anterior, and extensor digitorum longus muscles. Moreover, grasping strength and activity in the nocturnal phases were also sufficiently restored with VC supplementation. These results were similar to those previously reported for female SMP30-deficient mice. Thus, VC is essential for the maintenance of skeletal muscle function, and a skeletal muscle can recover reversibly upon VC supplementation, even if its function was once declined due to VC deficiency. These recovery abilities were observed to be similar in male as well as female SMP30-deficient mice.

The decrease in skeletal muscle weight due to VC deficiency may be due to an increase in reactive oxygen species in skeletal muscles [30,31,32]. We previously showed that reactive oxygen species were considerably increased in the skeletal muscles of female SMP30-deficient mice due to VC deficiency [26]. Furthermore, VC is necessary for the construction of collagen fibers [6,33,34,35]. In particular, since tendons that connect bone to muscles are rich in collagen, VC deficiency may cause collagen fibers to become brittle and unable to function, thus, leading to a decrease in skeletal muscle weight and physical ability.

## 5. Conclusions

In conclusion, VC-deficiency-induced muscle atrophy and declined physical ability were observed in male as well as female SMP30-deficient mice. These results indicated that VC is essential for maintaining skeletal muscle functions. Notably, the reduction in physical ability due to VC deficiency occurred earlier in female SMP30-deficient mice than in male SMP30-deficient mice. Even if skeletal muscle weight decreased due to VC deficiency, it could be recovered with VC supplementation. Therefore, VC could prove to be a useful treatment option for muscle atrophy. Finally, an adequate daily VC intake is necessary to avoid muscle atrophy and the loss of physical ability during aging.

## Data Availability

Data are included in the text; raw data are available from the corresponding author.
